# Lab-on-a-Chip Metabolic Analysis Using Benchtop NMR Technology

**DOI:** 10.1021/acs.analchem.5c04319

**Published:** 2026-01-19

**Authors:** Marc Azagra, Hetal Patel, Alejandro Portela, Dian Weerakonda, Behdad Aghelnejad, Jose Yeste, Gergő Matajsz, Marc Dubois, Matthew Fallon, Tryfon Antonakakis, Javier Ramón-Azcón, Irene Marco-Rius

**Affiliations:** † Institute for Bioengineering of Catalonia, Barcelona Institute of Science and Technology, 08028 Barcelona, Spain; ‡ Oxford Instruments, OX13 Abigndon, U.K.; § Multiwave Imaging, 13013 Marseille, France; ∥ Multiwave Technologies, 1228 Geneva, Switzerland

## Abstract

Organ-on-chip (OoC) systems are advancing rapidly as physiologically relevant in vitro models. However, real-time, noninvasive metabolic monitoring tools remain lacking. While NMR offers a powerful solution, current setups are not compatible with the planar format of microfluidic chips. Here we introduce the first benchtop NMR spectrometer for real-time metabolic monitoring of cell cultures on microfluidic platforms, utilizing hyperpolarization via dissolution dynamic nuclear polarization. This work details modifications made to a commercial benchtop NMR spectrometer, including the design and fabrication of a microfluidic platform that enables precise injection of hyperpolarized substrates and continuous renewal of cell culture media. The platform integrates a radiofrequency coil for signal transmission and reception and incorporates a custom-built sample carrier. Preliminary NMR data acquired with this system demonstrate its feasibility for dynamic metabolic studies.

## Graphical abstract

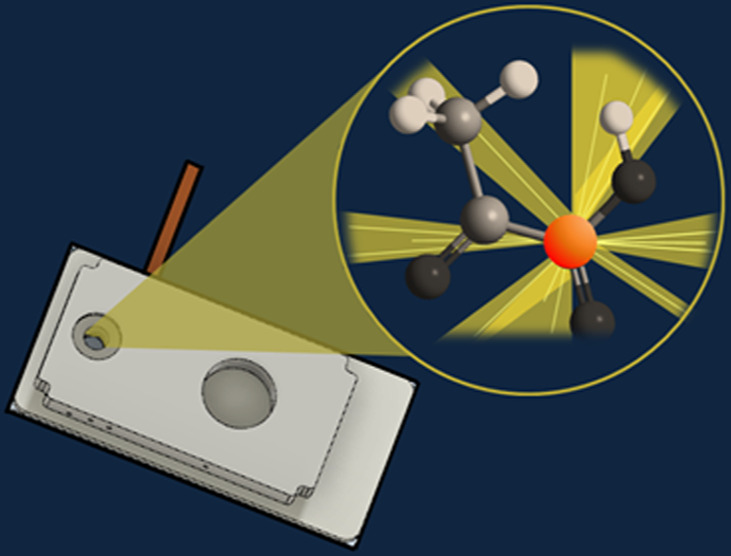

## Introduction

Benchtop NMR spectrometers (1.4–2 T) emerged as a practical and cheaper alternative to high field NMR spectrometres (3–28 T).^1^ These portable spectrometers employ permanent magnets, making them a cost-effective, compact, and cryogen-free system that eliminates the need for liquid nitrogen or helium refilling. Although their technical capabilities are limited, they are emerging as a viable option for use in locations and facilities with financial constraints, physical limitations, or restricted space availability.^2^ Their demand has been growing in a wide range of applications, such as material science,^3^ quantitative NMR for compound purity (assay or potency),^4,5^ food science,^6^ reaction monitoring processes,^7^ metabolomics,^8^ and hyperpolarized NMR methods development.^9^

The lower sensitivity and resolution of low-field NMR spectrometers, compared to high-field systems, have limited their application in metabolomics and real-time tracking of metabolic tracers. However, hyperpolarization techniques enhance the polarization of nuclear spins to levels far exceeding those achievable under thermal equilibrium conditions,^10^ which translates directly into more intense NMR signals.^11^ Recent advances in hyperpolarized NMR are driving the development of benchtop-compatible polarizers,^12^ which, when combined with compact NMR spectrometers, promise to make hyperpolarized NMR more accessible, affordable and versatile across a wide range of applications. While hyperpolarization mitigates sensitivity constraints, the use of ^13^C-labeled substrates offers significantly wider spectral dispersion compared to ^1^H NMR, reducing peak overlap and improving the resolution of individual metabolic pathways—an especially critical advantage for benchtop NMR systems, where limited field strength exacerbates spectral crowding in conventional ^1^H-based metabolomics.

Another emerging area of NMR research is in micro-NMR and microfluidic devices, particularly in analytical chemistry, where their ability to handle small sample volumes and integrate complex chemical, biological and physical processes offers significant advantages.^13^

Recognizing the potential of hyperpolarization and microfluidic chips, several studies have begun exploring the combination of both techniques using MRI scanners^11^ or high field spectrometers.^14–16^

Unfortunately, current commercial benchtop NMR spectrometer manufacturers offer 5 mm o.d. NMR tube probes, restricting compatibility with vessels of different shapes. Additionally, samples must be confined within an NMR tube and positioned at the bottom, where the spectrometer coil’s detection region is located.^17^

In this study, we designed and developed a benchtop NMR spectrometer and a microfluidic device optimized for real-time metabolic measurements using hyperpolarization techniques (Figure 1). While the system is compatible with any liquid-state hyperpolarization method, here we used dissolution dynamic nuclear polarization (dDNP) as a proof of concept. The microfluidic device incorporates a custom-designed X-nuclei transmitter/receiver (Tx/Rx) saddle radiofrequency (RF) coil to maximize signal-to-noise ratio (SNR) and *B*_1_ homogeneity within the microfluidic well, where the sample is analyzed. Furthermore, the coil is designed to generate a *B*_1_ field transverse to the *B*_0_ field of the permanent magnet, allowing the chip to be inserted flat and upright, thus minimizing potential issues related to gravity.

**Figure 1:**
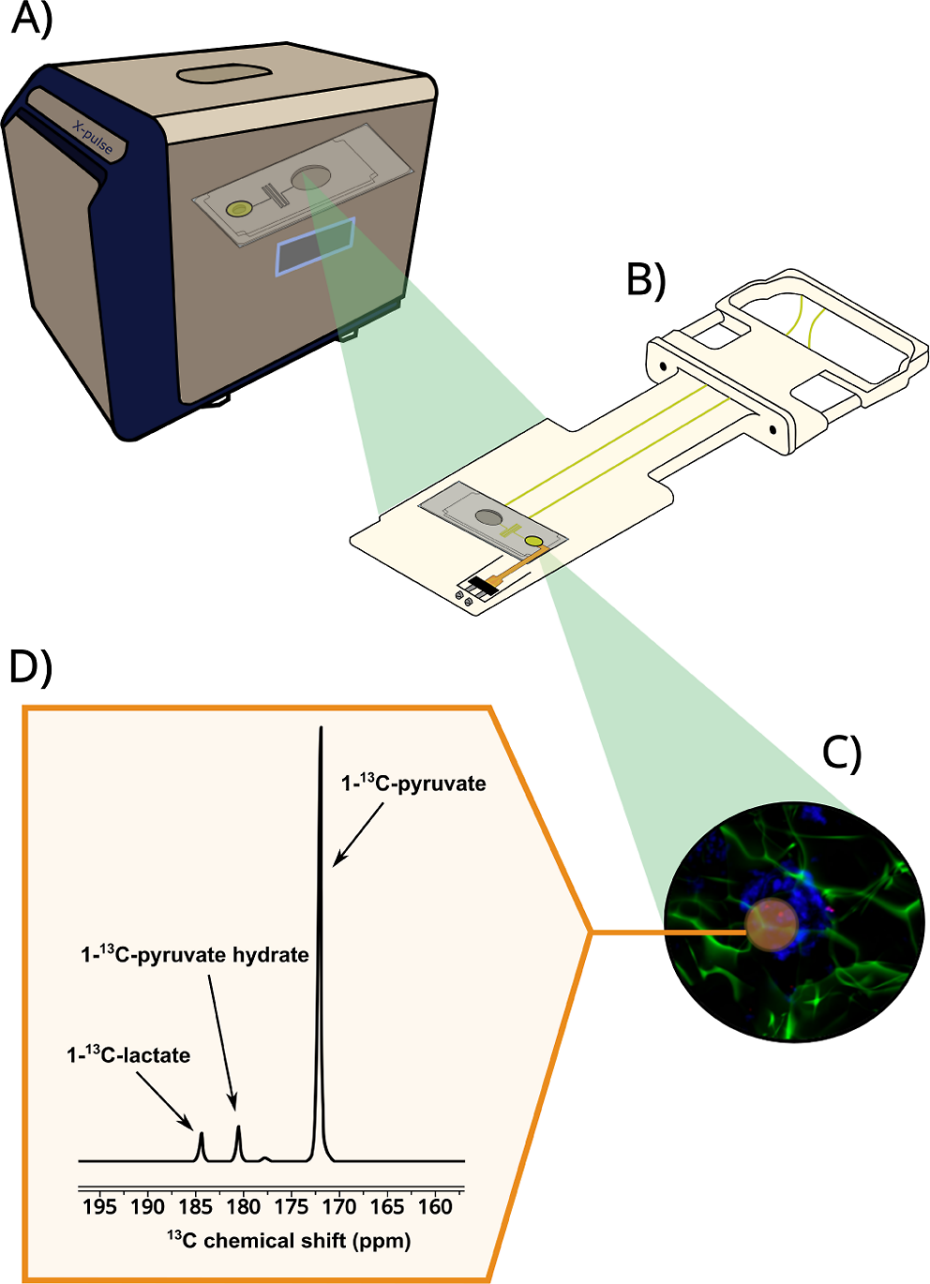
Overview of the design of benchtop NMR spectrometer for the detection of hyperpolarization-enhanced NMR metabolism in 3D tissue engineered cell models. (A) NMR spectrometer. (B) Chip carrier with the microfluidics chip and the embedded RF coil. (C) 3D liver cancer spheroids positioned within the chip’s detection region. (D) Example of ^13^C NMR spectrum observed upon injection of hyperpolarized [1-^13^C]­pyruvate and its conversion to lactate by the cancer cells in C.

This setup is versatile, accommodating a wide range of hardware variations, including coil design, size, shape, and material. Additionally, it enables testing of different platform shapes, sizes, and channel configurations, making it adaptable to various user requirements. For example, we developed a microfluidic platform with strong potential for organ-on-a-chip applications, where controlled flow and compartmentalization are essential to reproduce physiological conditions. Because it is compatible with benchtop NMR systems, it enables in-cell, noninvasive, real-time metabolic monitoring using hyperpolarisation-enhanced NMR, providing the sensitivity required to detect low-concentration biomarkers that are critical for assessing organ-level responses in vitro.^18^

To demonstrate the performance and usability of the system, we present measurements of the *T*_1_ of [1-^13^C]­pyruvate within the microfluidic device, as well as real-time metabolic experiments acquired using the custom-built saddle coil to assess system efficiency. In these proof-of-concept experiments, we mixed hyperpolarized [1-^13^C]­pyruvate with a solution containing lactate dehydrogenase (LDH) and nicotinamide adenine dinucleotide (NADH, enzyme cofactor), to catalyze the reduction of pyruvate to lactate via an enzymatic reaction. Under these conditions, we were able to determine the kinetic constant for the pyruvate-to-lactate conversion (*K*_PL_). Additionally, we tested cellular metabolism using a human liver carcinoma cell line (HepG2 cells) in suspension and measured the *K*_PL_ for this cell line. This study evaluates the performance of the entire hardware setup (chip, coil, and NMR spectrometer) for noninvasive, real-time metabolic studies of in vitro models.

## Methods

### Development of a Bencthop NMR Spectrometer for Lab-on-a-Chip Applications (BLOC Spectrometer)

A 1.4 T commercial benchtop NMR spectrometer (X-Pulse, Oxford Instruments) was modified to enable the insertion of planar microfluidic chips between the parallel magnets generating the *B*_0_ magnetic field. In this paper, this spectrometer is referred to as “the BLOC spectrometer”. To facilitate the correct insertion of the microfluidic chips, a carrier was 3D printed using photopolymer resin (Formlabs White V4 Resin) to hold the chip outside the spectrometer prior to the NMR experiment. This carrier also allowed for the connection of the corresponding capacitors to the saddle coil embedded in the microfluidic device for measurement (Figure 1).

### Microfluidic Chip Design and Fabrication

A microfluidic chip (Figure 2) was specifically designed and fabricated for this NMR spectrometer with the following requirements: (i) an experimental sample well to be placed in the homogeneous *B*_0_ region of the spectrometer, (ii) a reliable method to control the flow rate during the injection of hyperpolarized substrates, (iii) a constant delivery and renewal system for cell media, and (iv) integration of a Tx/Rx RF coil for ^1^H and ^13^C nuclei. The microfluidic chip was constructed from a glass substrate and a single layer of polydimethylsiloxane (PDMS, Sylgard 184, Dow Corning, USA), using a standard replica molding protocol and a computed numerical control (CNC)-milled poly­(methyl methacrylate) (PMMA) as a mold. The chip dimensions were 75 mm × 38 mm × 12 mm, with an average weight of approximately 20.25 g. Fluid handling was achieved through one main inlet channel that supplied the hyperpolarized solution to the well via a passive pump, two auxiliary inlets that delivered fresh medium to the bottom of the well, and one outlet positioned at midheight that facilitated renewal of the well solution. The passive pump consisted of an elastomeric cavity of 15 mm in diameter and 200 μm in height covered by an 800 μm-thick PDMS membrane and connected to the well through a serpentine microfluidic channel 300 μm in width, 200 μm in height, and 15 mm in length. This combination of membrane cavity and microfluidic channel resistance, which could be optimized during design, enabled controlled, repeatable administration for the NMR detection. The sample well was sealed on top by a removable round cover glass, which allowed for easy access to the cell chamber and enabled sealing once the cells or engineered tissue had been inserted.

**Figure 2:**
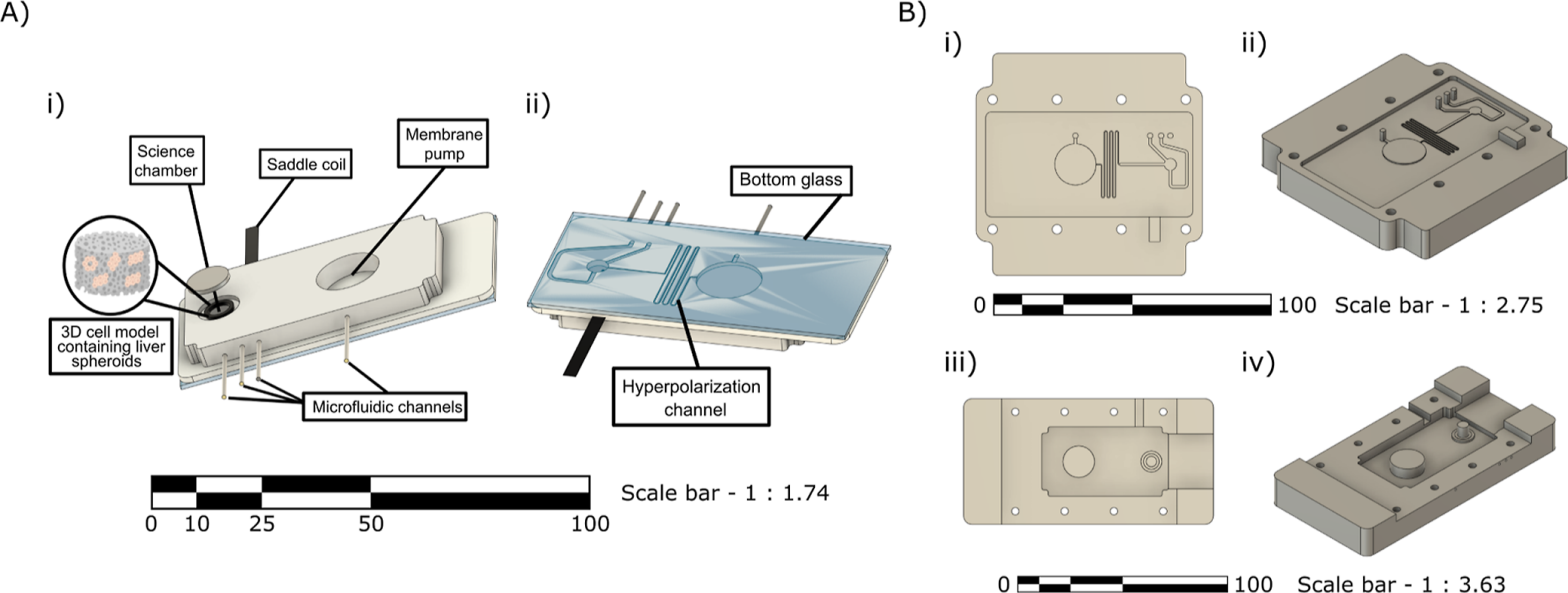
Microfluidic device and fabrication mold. (A) (i) Tilted top view of the microfluidic device highlighting key features, including the membrane pump, microfluidic channels, saddle coil embedded around the sample well, the scientific chamber (well), and the expected engineered 3D cell model inserted into the well. (ii) Bottom view of the microfluidic device showing the hyperpolarization channel and the bottom glass. (B) Poly­(methyl methacrylate) (PMMA) mold used to fabricate the microfluidic device. (i) Top view of the mold replicating the bottom side of the chip. (ii) Lateral view of the mold replicating the bottom side of the chip. (iii) Top view of the mold replicating the top side of the chip. (iv) Lateral view of the mold replicating the top side of the chip.

### Integration of an RF Coil into the Microfluidic Chip

The design of the saddle coil was optimized numerically with CST studio suite (Electromagnetic wave solver, Dassault Systemes, France). A uniform cylinder representing the scaffold dimensions (height 2 mm and diameter 5 mm) was placed in the center of the saddle coil (relative permittivity 80 and conductivity 1.4 T) to load the probe and evaluate the coil sensitivity in the volume of interest. The coil was placed within a gap of 8.4 mm with metallic plates on upper and lower surfaces acting as the ground planes present in the spectrometer for RF shielding. Several parameters such as the number of turns, the height and the diameter of the saddle coil were optimized to reach the highest average *B*_1_ amplitude within the scaffold volume. Coil performance was evaluated at 15 MHz, corresponding to the ^13^C Larmor frequency at 1.4 T. The *B*_1_ field normalized to an input power of 1 W was quantified during each step of the optimization process. The copper traces of the final coil design were printed on polyimide substrate with two layers of 35 μm-thick copper. The coil was produced with Eagle (Computer Aided Design software for printed circuit board, Autodesk Inc., USA), two vias were placed to connect the two copper layers. The saddle coil winding was printed on the inner surface and the feed with the connector were printed on the outer surface. Additional polyimide layers were added to avoid contact between the sample liquid and the copper traces. The final coil was 215 μm-thick. The coils were printed flat and manually formed into cylinder before being embedded into the microfluidic chip.

### Hyperpolarization of [1-^13^C]­pyruvate via dDNP

To hyperpolarize [1-^13^C]­pyruvate, a solution containing 24 μL of [1-^13^C]­pyruvic acid (Merck, Darmstadt, Germany), 15 mM trityl radical OX063 (GE Healthcare, Illinois, U.S.A.) and 1.5 mM gadoteric acid (Guerbet, Villepinte, France) was placed into a sample cup and inserted into a dDNP polarizer (HyperSense, Oxford Instruments Ltd., Oxford, U.K.) operating at 3.35 T. This sample was cooled down to 1.3 K, and irradiated with 100 mW microwaves at 94.115 GHz for approximately 40 min. The hyperpolarized frozen solid was then rapidly dissolved and flushed out of the polarizer by injecting a heated phosphate buffered saline solution (PBS) supplemented with 1% 4-(2-hydroxyethyl)-1-piperazineethanesulfonic acid (HEPES), 0.01% ethylenediaminetetraacetic acid (EDTA), 0.1% sodium chloride (NaCl), and 0.2% sodium hydroxide (NaOH) (pH 12) to yield a 5 mL solution of 80 mM [1-^13^C]­pyruvate at pH 7 and around 12.5% ^13^C polarization at the time of dissolution,^19^ which was collected in a 50 mL plastic falcon tube. Details of the polarization measurement are provided in the Supporting Information document and in ref 19.

### *T*_1_ Measurement of Hyperpolarized [1-^13^C]­pyruvate at 1.4 T

Immediately after dDNP, the resulting hyperpolarized pyruvate solution was injected either into the microfluidic chip via the membrane pump channel for *T*_1_ measurements using the 1.4 T BLOC spectrometer (3 s from the start of injection to complete filling of the science chamber), or into a 5 mm o.d. tube for measurements with a commercial 1.4 T bencthop NMR spectrometer (Pulsar, Oxford Instruments). In both cases, the acquisition parameters were as follows: repetition time (TR) = 4 s, acquisition points = 16,384, ^13^C offset = 6190 Hz, 30° flip angle, receiver attenuation = 28 dB, WALTZ decoupling, and probing temperature = 37 °C. The longitudinal relaxation time *T*_1_ was obtained by integrating the C1 resonance of [1-^13^C]­pyruvate over time, fitting the decay to a monoexponential function and applying a correction for excitation-induced magnetization loss according to the flip angle used^19^

$$y=A+M_{0}e^{-t/T_{1,app}}$$ 1

2 $$T_{1}={\left(\frac{1}{T_{1,app}}+\frac{\ln\left(\cos\alpha \right)}{TR}\right)}^{-1}$$

### Real-Time Assessment of Pyruvate to Lactate Conversion Catalyzed by LDH Using the BLOC Spectrometer

A stock solution containing 1:100 dilution of LDH, 33 mM NADH and in 600 μL of PBS buffer was prepared and kept at 37 °C.

Immediately after the ejection of the hyperpolarized solution from the dDNP polarizer, 8 μL of an 80 mM hyperpolarized [1-^13^C]­pyruvate solution was mixed with 120 μL of the LDH-containing solution in a 1.5 mL Eppendorf tube. The resulting mixture was rapidly injected through the injection channel of the microfluidic chip, which had already been inserted in the BLOC spectrometer, and NMR acquisition commenced. A series of NMR spectra were acquired using the same experimental parameters as described above. Pyruvate-to-lactate conversion was detected, and the corresponding *k*_PL_ rate constant was quantified from the acquired data (see the Data Analysis and Kinetic Rate Calculation section).

NMR data were obtained using the BLOC spectrometer and its X-pulse Spinflow software. The *B*_0_ inhomogeneities were corrected with an optimized shimming protocol using a microfluidic device filled with water prior to the cell-containing chip insertion.

### Real-Time Metabolic Analysis of HepG2 Cells Using the BLOC Spectrometer

All cell culture reagents were obtained from Thermofisher Scientific-Gibco unless stated otherwise. HepG2 human hepatocarcinoma cells (CliniSciences S.L.) were cultured in Eagle’s Minimum Essential Medium (EMEM, ATCC) supplemented with 10% fetal bovine serum (FBS) and 1% penicilin-streptomycin (P/S) (10^4^ U mL^–1^). Cells were cultured and maintained in 175 cm^2^ flasks and incubated at 37 °C under 5% CO_2_. Cell media was changed every 48–72 h and cells passaged at 80% confluence. Immediately before the hyperpolarized NMR acquisition, the flask was washed with PBS, cells were detached using Trypsin–EDTA 0.25% and centrifuged for 3 min at 200 g. Cell viability and count were assessed by trypan blue staining. A total of 10^7^ cells were transferred into a 1.5 mL Eppendorf tube containing 120 μL of EMEM medium, with the temperature maintained at 37°. Immediately after the ejection of the hyperpolarized solution from the dDNP polarizer, 8 μL of hyperpolarized [1-^13^C]­pyruvate solution was added to the cell-containing Eppendorf. The resulting mixture was manually pipetted without delay into the scientific chamber of the microfluidic chip. After sealing the well with a round glass cover, the carrier was inserted in the spectrometer, and NMR acquisition started - by which time the cells had been in contact with the hyperpolarized pyruvate solution for ≈25–30 s.

The NMR data acquisition and processing were identical to those described in the previous sections.

### Data Analysis and Kinetic Rates Calculation

MestreNova 15.0.0 was used for NMR data processing. To analyze the reaction kinetics catalyzed by LDH, a widely used simplified mathematical model to fit the corresponding kinetic constants was employed.^20–23^ This model considers only the conversion of the hyperpolarized substrate (pyruvate) to the product (lactate) at a constant rate (*K*_PL_), the reverse reaction at a constant rate (*K*_LP_), and the signal loss of both the substrate and product due to their corresponding apparent longitudinal relaxation (*T*_1,app_). The differential equations governing these reactions are as follows

3 $$\frac{d\left[Substrate\right]}{dt}=K_{LP}\left[Product\right]-\left(\frac{1}{T_{1,app}^{Substrate}}+K_{PL}\right)\left[Substrate\right]$$

4 $$\frac{d\left[Product\right]}{dt}=K_{PL}\left[Substrate\right]-\left(\frac{1}{T_{1,app}^{Product}}+K_{LP}\right)\left[Product\right]$$

We implemented the parameter fitting code using the Julia programming language, considering only the data corresponding to metabolic products. The differential equations were solved numerically using an initial value problem (“IVP”) solver for stiff systems (“CVODE BDF”). For the optimization process, we employed the mean squared error (MSE) as the cost function, defined as follows

5 $$MSE=\frac{1}{N}\sum_{i=1}^{N}{\left(y_{i}-{ŷ}_{i}\right)}^{2}$$

were *N* represents the total number of data points, *y*_*i*_ the experimental data and ${ŷ}_{i}$ the simulated one. We solved the optimization problem using a global optimizer (i.e., Differential Evolution) from the Julia package BlackBoxOptim.jl., setting reasonable bounds for all the parameters and allowing the optimization to run for a maximum of 3 min (convergence of the results for all optimizations happened between 30 s and 2 min).

## Results and Discussion

### Design of a Benchtop NMR Spectrometer (BLOC Spectrometer) and a Compatible Microfluidic Chip for Lab-on-a-Chip Applications

We modified a 1.4 T commercial benchtop NMR spectrometer to enable the integration and study of planar microfluidic chips. The system counts with a *B*_0_ homogeneous region of 5 mm^2^ (Figure 1).

We fabricated a PDMS-based microfluidic chip (Figure 2), featuring 300 μm in width and 200 μm in height channels connected to a cylindrical chamber (5 mm × 5 mm) designed for NMR detection (referred to as “scientific chamber” in this work). This setup included a passive membrane pump engineered to transport hyperpolarized solutions. With each manual injection of hyperpolarized solution, the membrane cavity first inflated storing the liquid; then, as it deflated, it administrated the solution to the scientific chamber in a repetitive manner at a defined time and flow profile. This controlled delivery reduced pressure surges during hyperpolarized solution injections. The selected use of PDMS and glass materials minimized susceptibility artifacts during measurement. Following geometric optimization, we designed and fabricated a saddle RF coil, featuring a 6-turn structure with a height of 5.4 mm a diameter of 5.5 mm. The copper tracks measured 0.27 mm in width and 0.035 mm in thickness. Figure 3 presents the *B*_1_ field amplitude maps for transversal, sagittal and coronal planes. The average *B*_1_ amplitude within the scaffold volume was determined to be 1.16 mT at 15 MHz for a 1 W power input. For comparison, simulations of a standard 5-turn solenoid probe used in the commercial Pulsar benchtop (Oxford Instruments) configuration yielded an average *B*_1_ field of 1.32 mT under identical conditions. Within the scaffold volume, the *B*_1_ field homogeneity exhibited a relative standard deviation of 23%, which is higher than the 4.5% achieved with the 5-turn solenoid probe.

**Figure 3:**
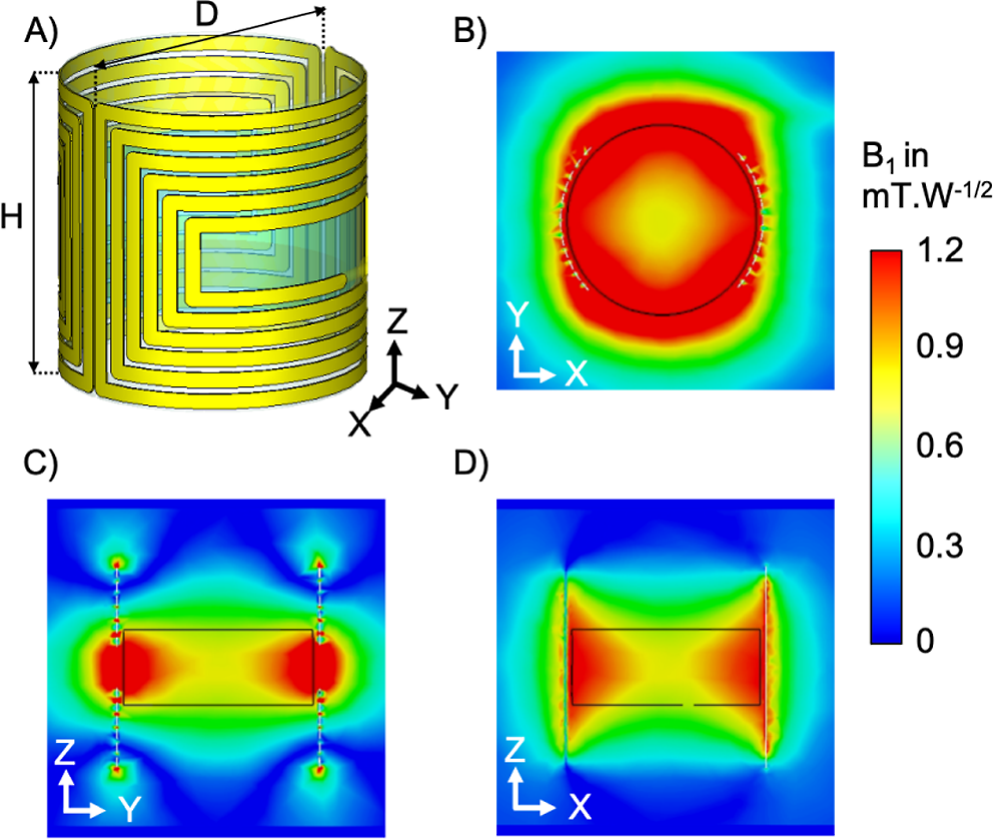
Simulation results for the optimized saddle coil: (A) schematics of the 2 mm height coil, 6 turns, 0.6 mm diamameter, relative permittivity 80 and conductivity 1.2 S m^–1^, (B) *B*_1_ field magnitude in the transversal plane, (C) *B*_1_ field magnitude in the coronal plane, (D) *B*_1_ field magnitude in the sagittal plane. The black thin line represents the cylindrical phantom position.

We designed and built a custom RF coil dual-tuned to the ^1^H and ^13^C resonance frequencies, with the matching circuit illustrated in Figure 4. We evaluated various shimming methods, including 3D spatial field mapping analogous to gradient shimming, resulting in a line width below 6 Hz.

**Figure 4:**
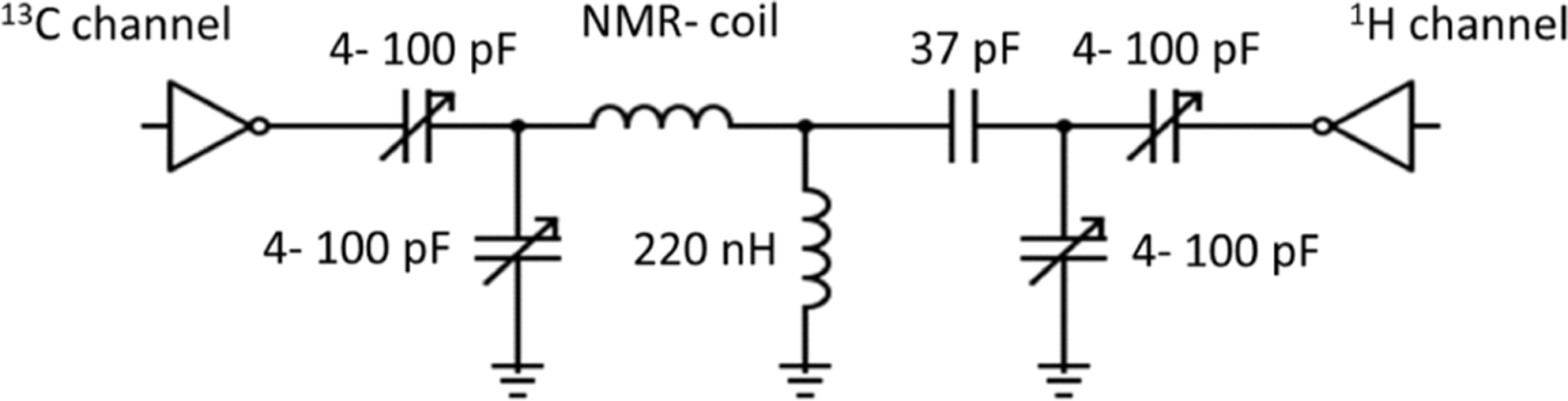
Schematics of the tuning and matching circuit used to interface the embedded RF coil with the spectrometer.

We experimentally validated the *B*_1_ efficiency of the saddle coil in the BLOC spectrometer setup. Results indicate that the 90° pulse duration was 16 μ s for the solenoid coil in the commercial NMR spectrometer and 18 μ s for the saddle coil in the BLOC spectrometer under identical experimental conditions at 15 MHz. The ratio of the simulated *B*_1_ amplitude, averaged over the scaffold volume, closely matched the inverse ratio of the measured 90° pulse duration, with an accuracy within 1%, demonstrating excellent agreement between simulations and experimental data. The ^13^C nutation curve for the BLOC spectrometer is shown in Figure S1 of the Supporting Information document.

### *T*_1_ Measurement of Hyperpolarized ^13^C Pyruvate at 1.4 T

We performed comparative experiments to evaluate the performance of the BLOC spectrometer against a commercial Pulsar NMR system. We first hyperpolarized [1-^13^C]­pyruvate using dDNP. In the BLOC spectrometer experiments, we injected the hyperpolarized pyruvate solution into the microfluidic chip through the membrane pump channel (Figures 5 and 6A). In contrast, for the Pulsar spectrometer, we injected the hyperpolarized solution into a 5 mm o.d. NMR tube.

**Figure 5:**
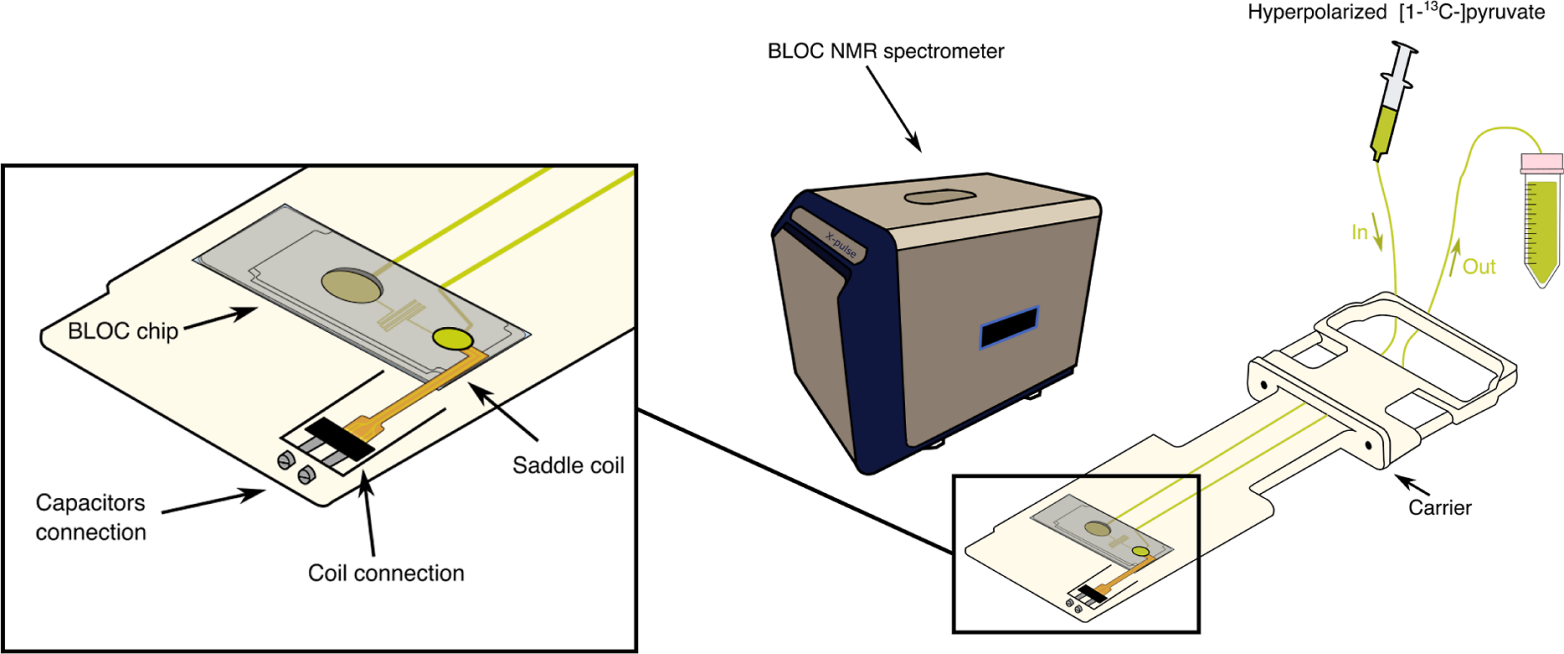
Schematic representation of the setup showing the injection of a hyperpolarized solution through the channels of the microfluidic device, which is positioned inside the spectrometer.

**Figure 6:**
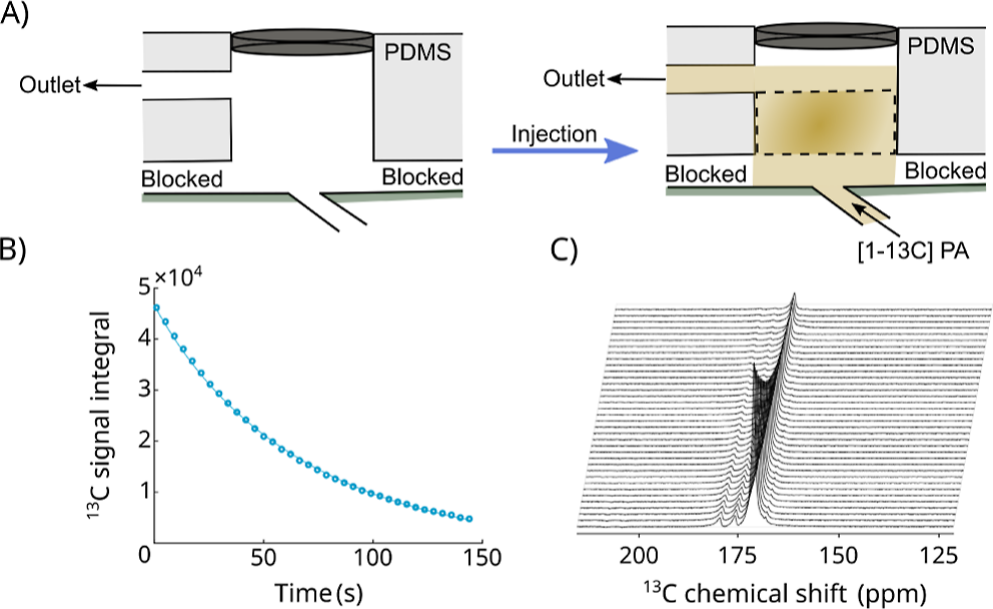
Longitudinal relaxation measurements of [1-^13^C]­pyruvate in the BLOC spectrometer setup. (A) Schematic of the procedure used to inject the hyperpolarized pyruvate dissolution into the microfluidic device through the hyperpolarization channel, filling the well. Two channels were blocked, leaving one inlet and one outlet. The dashed line in the right figure indicates the physical position of the saddle coil. (B) Plot illustrating the exponential decay of the [1-^13^C]­pyruvate signal due to *T*_1_ relaxation and radiofrequency pulses. The *T*_1_ value was calculated by fitting the exponential decay function, yielding a value of 72 ± 3.4 s. The stacked spectra represent the dynamic ^13^C measurement of hyperpolarized [1-^13^C]­pyruvate over time.

A ^13^C spectrum was acquired every 4 s over a total duration of 144 s, and the stacked spectra of all acquisitions are shown in Figure 6. We repeated this experiment three times. Using the BLOC spectrometer, we measured *T*_1_ values of 71.4, 76.8, and 69.1 s, yielding an average *T*_1_ of (72.4 ± 3.9 s) for the C1 carbon of [1-^13^C]­pyruvate at 1.4 T and 37 °C.

As expected, the results are consistent with those obtained using the commercially available benchtop NMR system operating at the same temperature and magnetic field strength, which yielded a *T*_1_ of 77 s. These values are, in turn, higher than the *T*_1_ of [1-^13^C]­pyruvate measured at the Earth’s magnetic field (56 s),^24^ which determines the polarization decay during sample transport (see Supporting Information).

When using the BLOC spectrometer setup, the full-width at half-maximum (fwhm) of the ^13^C signal was 13 ± 6 Hz, i.e. approximately 11–12 Hz broader than the line widths obtained with the commercial instrument using a standard 5 mm NMR tube probe.

### Real Time Metabolic Reaction Monitoring Using the BLOC Spectrometer Setup

We successfully observed the metabolic conversion of [1-^13^C]­pyruvate to [1-^13^C]­lactate in both enzymatic solutions and 2D cell cultures using the BLOC spectrometer setup. We performed experiments by mixing hyperpolarized [1-^13^C]­pyruvate with a solution containing LDH and NADH as the cofactor for the reduction reaction. We injected the hyperpolarized solution through the microfluidic chip’s injection channel, with the chip already placed inside the BLOC spectrometer (procedure described in Figure 5), and started the NMR acquisition immediately, with a delay between dissolution and start of acquisition of 25–30 s.

We acquired a series of 35 spectra over 100 s using a 30° flip angle, and detected the conversion of pyruvate to lactate. The kinetic rate constant *K*_PL_ for this enzymatic reaction was 7.82 × 10^–3^ s^–1^.

For the subsequent experiments, we used HepG2 cells in 2D cultures to study the pyruvate-to-lactate conversion. Injecting cells through the microfluidic channels often caused clogging and reduced the number of cells in the detection region, lowering the SNR. To overcome this, we adopted an alternative loading approach. We mixed the cells with the hyperpolarized [1-^13^C]­pyruvate solution, transferred the mixture into the microfluidic chip, sealed the well with a cover glass, and rapidly inserted the chip into the BLOC spectrometer. We repeated this procedure three times, with 8 μL of 80 mM [1-^13^C]­pyruvate in 120 μL of HepG2 cell media resulting in a 5 mM solution. A acquired a series of 10 spectra, one every 4 s, with a 30° flip angle each. In this case, the delay between dissolution and start of NMR acquisition was again about 30 s.

On average, the fwhm of the [1-^13^C]­pyruvate peak in the first spectrum was of 8.0 ± 6.1 Hz, with a SNR of 86.54. The first experiment showed a larger fwhm value (15.1 Hz) due to the presence of bubbles. In the subsequent two experiments, the fwhm were 4.0 and 4.1 Hz, respectively, with no bubbles observed.

The *K*_PL_ values calculated for the three experiments were 3.57 × 10^–3^ s^–1^ (Figure 7B), 3.08 × 10^–3^ s^–1^ (Figure 7D), and 3.55 × 10^–3^ s^–1^ (Figure 7F). The standard deviation across all three experiments was 2.7 × 10^–4^ s^–1^, demonstrating high reproducibility among all three experiments despite the presence of bubbles in the first experiment. The *k*_LP_ values were more than 10 orders of magnitude lower than the *k*_PL_ values, indicating that the reaction proceeded predominantly from pyruvate to lactate, with negligible back conversion.

**Figure 7:**
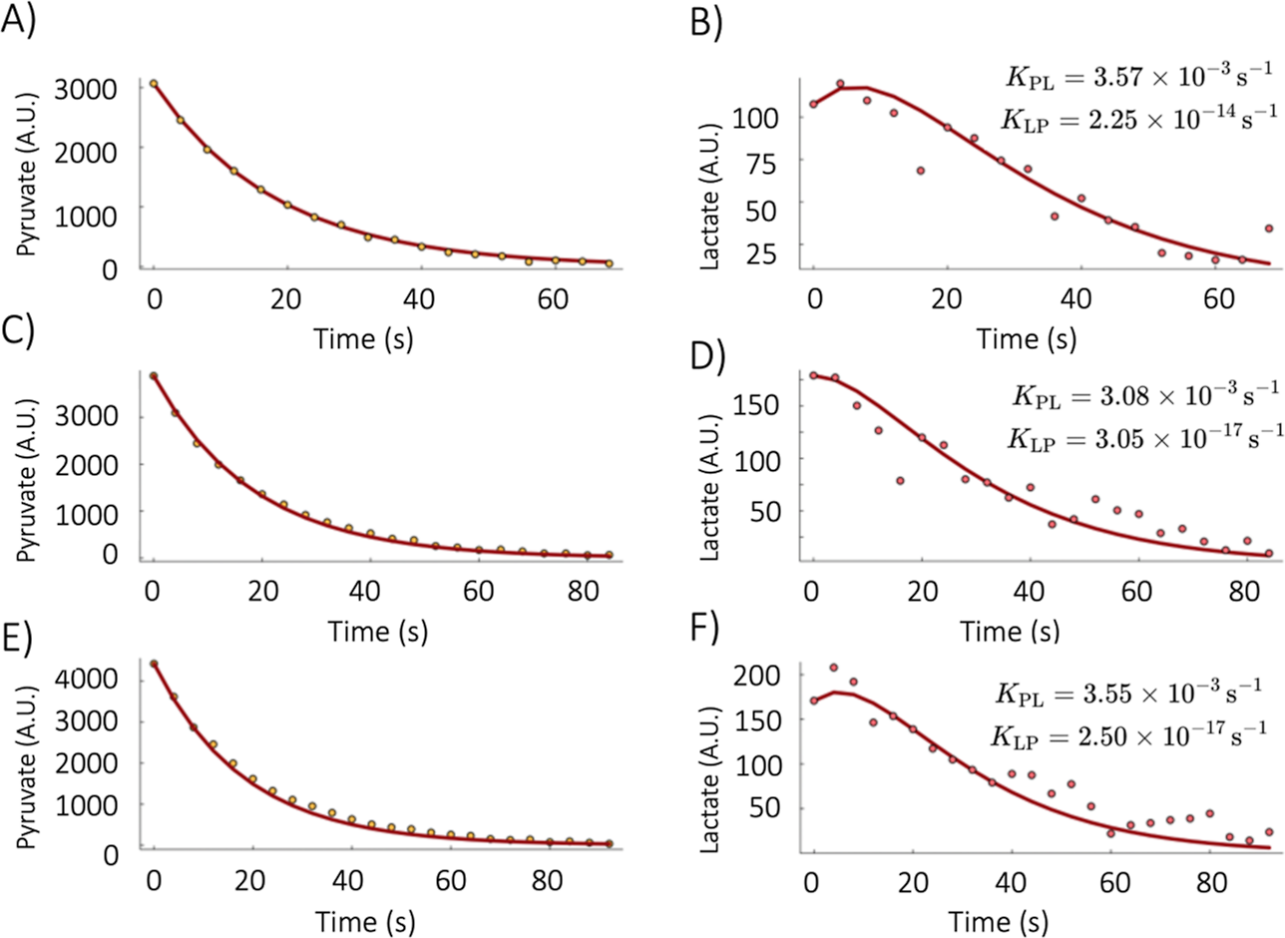
Kinetic analysis of HepG2 cell metabolism. Time 0 s corresponds to the start of NMR acquisition. The delay between the initial contact of the cells with the hyperpolarized substrate and the first acquired spectrum was accounted for in the kinetic rates calculations. (A) Monoexponential decay function fit to the [1-^13^C]­pyruvate polarization decay due to *T*_1_ relaxation, radiofrequency pulses, and metabolism for the first replicate. (B) Kinetic function fit to the production of [1-^13^C]­lactate for the first replicate. (C) Same as (A) for the second replicate. (D) Same as (B) for the second replicate. (E) Same as (A) for the third replicate. (F) Same as (B) for the third replicate.

The *K*_PL_ values obtained from the enzymatic reaction and the cells in suspension have the same order of magnitude and also compared with the ones found in the literature.^22,25^ The apparent *K*_PL_ in hyperpolarized ^13^C NMR reflects both enzymatic conversion and substrate transport, and depends on several biological and experimental factors. Cell type, density and pyruvate concentration influence metabolic activity, while oxygenation, pH, and nutrient conditions further modulate LDH function. Instrumental parameters such as field strength, pulse sequence and acquisition timing may also affect the measured *K*_PL_ values.

## Conclusions

In this study, we evaluated the integration of a modified benchtop NMR spectrometer with a cost-effective microfluidic platform for hyperpolarized-NMR measurements. We tested a microfluidic chip equipped with an X-nuclei transmitter/receiver saddle radiofrequency coil. Initially, we used the setup to measure the longitudinal relaxation constants of hyperpolarized [1-^13^C]­pyruvate via dDNP. Subsequently, we assessed real-time metabolic processes with enzymatic dissolutions and later with cells in suspension. This study demonstrates the potential for adapting hardware to meet specific user needs in hyperpolarized NMR experiments, including modifications to the spectrometer, coil, and microfluidic chip. The findings have broad implications for NMR applications, including coil testing, hardware development, and real-time metabolic analysis for drug testing. Future improvements in spectral resolution and reproducibility could be achieved by upgrading to advanced polarizers for higher sensitivity, automating substrate injection to minimize variability and polarization loss, and increasing cell density to enhance metabolic signals (although the latter may compromise the chip’s miniaturization benefits).

## Supplementary Material



## Notes

This sections includes: I. ^13^C nutation experiment for determination of the 90° pulse length (P90) II. Experimental conditions to measure ^13^C polarization (PDF)

## a

Aix Marseille Univ, CNRS, Centrale Med, Institut Fresnel, Marseille, France
