# Supplementary material for: Lab-on-a-Chip Metabolic Analysis Using Benchtop NMR Technology
Source: Anal Chem. 2026 Jan 19;98(4):2701–8. doi: 10.1021/acs.analchem.5c04319 (PMC12874212; doi:10.1021/acs.analchem.5c04319)
Supplement: Supplementary file 1 [file ac5c04319_si_001.pdf]

SUPPLEMENTARY INFORMATION FOR

Lab-on-a-Chip Metabolic Analysis Using Benchtop NMR Technology

Marc Azagra,[†] Hetal Patel,[‡] Alejandro Portela,[†] Dian Weerakonda,[‡] Behdad Aghelnejad,[‡] Jose Yeste,[†] Gergő Matajsz,[†] Marc Dubois,[¶] Matthew Fallon,[‡] Tryfon Antonakakis,[§] Javier Ramon-Azcon,[†] and Irene Marco-Rius[*,†]

[†] Institute for Bioengineering of Catalonia, Barcelona Institute of Science and Technology, 08028

Barcelona, Spain

[‡] Oxford Instruments, OX13 Abigndon, United Kingdom

[¶] Multiwave Imaging, 13013 Marseille, France

[§] Multiwave Technologies, 1228 Geneva, Switzerland

CONTENT

I. ^{13}C nutation experiment for determination of the 90° pulse length (P90)
II. Experimental conditions to measure ^{13}C polarization

I. P90 value calculation

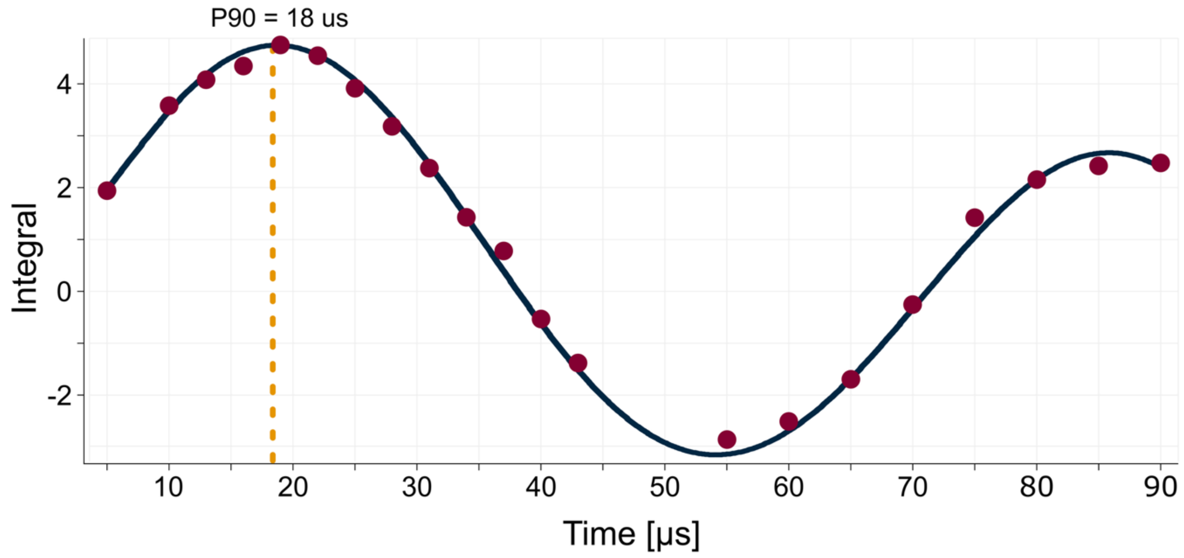

Figure S1. ^{13}C nutation curve showing the dependence of signal integral on pulse length, where the maximum corresponds to the optimal 90° pulse (P90).

II. Experimental conditions to measure ^{13}C polarization

The thermal measurement was performed immediately after the hyperpolarized experiment, using the same sample to ensure consistency.

The polarization was calculated using the following formula derived from the Boltzmann distribution:

$$P_{HP} = \frac{I_{HP}/(N_{HP} \cdot \sin(\theta_{HP}))}{I_{Thermal}/(N_{Thermal} \cdot \sin(\theta_{Thermal}))} \cdot \frac{\gamma \hbar B_0}{2k_B T}$$

Where:

- I_{HP} and $I_{thermal}$ are the integrated NMR signal intensities of the hyperpolarized and thermal measurements, respectively.
- N_{HP}, $N_{thermal}$: Number of scans used in each experiment.
- θ_{HP}, $\theta_{thermal}$: Flip angles used in each experiment (in radians).
- γ is the gyromagnetic ratio of 13C.
- $\hbar$ is the reduced Planck constant.
- B_0 is the magnetic field strength.
- k_B is the Boltzmann constant.
- T is the absolute temperature during acquisition.

This approach allows for a direct estimation of the absolute polarization level in solution at the first acquisition of the hyperpolarized experiment. Then, the actual polarization value at the time of dissolution was refined by backcalculating the expected exponential decay due to longitudinal relaxation. This correction accounts for polarization losses occurring during sample transfer and setup prior to measurement. For example, given that we measured the T_1 at the Earth's magnetic field to be approximately 56 s,[1] a transfer time of 25 s would reduce the polarization from 12.5 % at dissolution to about 8 % at the time of the first acquisition.

References:

[1] Eills, J.; Azagra, M.; Gómez-Cabeza, D.; Tayler, M. C.; Marco-Rius, I. Polarization losses from the nonadiabatic passage of hyperpolarized solutions through metallic components. *Journal of Magnetic Resonance Open* 2024, 18, 100144.
